# A Study on Doping and Compound of Zinc Oxide Photocatalysts

**DOI:** 10.3390/polym14214484

**Published:** 2022-10-23

**Authors:** Tan Mao, Mengchen Liu, Liyuan Lin, Youliang Cheng, Changqing Fang

**Affiliations:** 1 College of Mechanical and Material Engineering, North China University of Technology, Beijing 100144, China; 2 College of Printing, Packaging and Digital Media, Xi’an University of Technology, Xi’an 710000, China

**Keywords:** zinc oxide, photocatalysis, doping, compound

## Abstract

As an excellent semiconductor photocatalyst, zinc oxide is widely used in the field of photocatalysis and is regarded as one of the most reliable materials to solve environmental problems. However, because its band gap energy limits the absorption of visible light and reduces the efficiency of catalytic degradation, it needs to be doped with other substances or compounded with other substances and precious metal. This paper summarizes the research on this aspect at home and abroad in recent years, introduces the doping of transition metal ions by zinc oxide, the compounding of zinc oxide with precious metals or other semiconductors, and the prospect of further improving the catalytic efficiency of zno photocatalyst is also put forward.

## 1. Introduction

With the development of the times and the development of science and technology, such as textile, printing and dyeing, paint, medicine and other fields of industrial production will produce a large number of industrial wastewater discharged into the surrounding water, resulting in serious pollution [1,2,3]. The large number and unpredictable composition of organic dyes undoubtedly increase the difficulty of treatment. Traditional industrial wastewater treatment methods include flocculation-flocculation [4,5], electroflocculation [6], flocculation-carbon adsorption [7], electrochemistry, advanced oxidation, and biochemistry, etc.; however, these methods have some problems, such as high energy consumption, high cost, and unsatisfactory treatment effect. The semiconductor composites use light energy to create hole–electron pairs and use the electron holes in the surface active sites as reducing and oxidizing agents to promote redox, respectively. Therefore, the organic pollutants in sewage can be completely decomposed into inorganic small molecules, which has the characteristics of continuous and non-secondary pollution, and has been widely concerned. As a result, the inorganic semiconductor photocatalyst in recent years in the field of organic pollutants in the degradation of the important application prospects of attention [8,9,10,11].

Among the various photocatalysts, TiO_2_ that pass for the most widely employed “golden ”, photocatalyst has been largely used in heterogeneous photocatalysis, due to its chemical stability, nontoxicity [12,13,14]. Compared with titanium dioxide, zinc oxide has been found to have higher quantum efficiency and theoretical photocatalytic efficiency than titanium dioxide and it is a promising photocatalyst [15,16,17]. The photocatalytic degradation mechanism of zinc oxide is similar to titanium dioxide [18,19], so zinc oxide is regarded as a possible alternative to titanium dioxide. Some studies have shown that zinc oxide has a higher photocatalytic efficiency than titanium dioxide in degrading some hard-to-degrade organic compounds [20,21,22,23]. Zinc oxide is one of the important semiconductor materials of the II–VI family, which belongs to n-type semiconductor. Due to its excellent thermal stability and non-toxic properties, zinc oxide has been widely used in coatings, electronics, photocatalysis and sensor fields. In the field of photocatalysis [24,25], the band-gap energy of zinc oxide is 3.37 ev, which limits the most efficient catalytic degradation of zinc oxide under UV light and is less efficient under natural visible light. However, in the actual working environment, the continuous use of UV lamps is not as good as the use of natural visible light from the perspective of cost and environmental protection. Because the band gap energy of zinc oxide determines that it can only use the ultraviolet part of sunlight and the pimping proportion of ultraviolet light in natural light which is only 4%, the catalytic degradation efficiency of zinc oxide under visible light is not high. In order to improve the photocatalytic performance of zinc oxide, many attempts have been made, such as refining the grain size of zinc oxide [26], increasing the specific surface area [27,28], controlled microwave-assisted and pH-affected growth of zinc oxide [29] etc. At present, the most efficient method is element doping and recombination with precious metals or other semiconductors to improve the light response range of zinc oxide.

**Figure 1:**
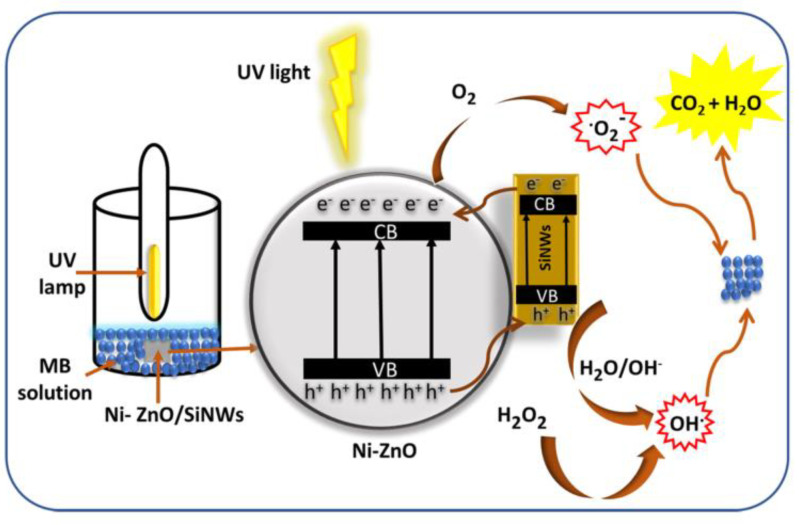
Photocatalysis of zinc oxide.

The specific degradation mechanism of zinc oxide is shown in the Figure 1. When zinc oxide absorbs light energy, the electrons transition from the valence band to the conduction band, because creating a photogenerated electron–hole pair in the zinc oxide crystal, electrons combine with adsorbed oxygen to form superoxide radicals, and holes combine with water molecules to form hydroxyl radicals, finally, highly oxidizing hydroxyl and superoxide radicals react with substances that need to be degraded, such as methyl blue and tetracycline, to turn them into harmless water and carbon dioxide. In this paper, several methods to improve the photocatalytic performance of zinc oxide are summarized, examples are given to illustrate how different methods improve the photocatalytic performance of zinc oxide, and the specific mechanism of different methods to improve the photocatalytic efficiency of zno was introduced. The purpose of the summary is to compare several different methods of analogy, some new thinking and then get some new ideas can better improve the efficiency of zinc oxide photocatalysis. Through this summary, it is found that all kinds of methods in the field of photocatalysis of zinc oxide have certain limitations, so we should break the rules and broaden the common methods and ideas to prepare the zinc oxide photocatalytic material with better performance.

The photocatalysis of zinc oxide is not only widely used to degrade pollutants but also widely used in water decomposition and CO_2_ reduction. In the field of water decomposition and CO_2_ reduction, as applied to the degradation of pollution aspects of the same trouble, the wide band gap (3.3 eV) of ZnO semiconductor can only absorb the ultraviolet spectrum (<400 nm), which accounts for 3–5% of the solar spectral radiation, which seriously limits its utilization of solar energy. In addition, the photogenerated electrons and holes generated on ZnO semiconductor materials are prone to recombination, and the surface water oxidation kinetics are slow, which further reduces the utilization rate of solar energy and seriously limits the practical application of ZnO semiconductor materials. At present, the main solutions include element doping, quantum dot sensitization, noble metal deposition, heterostructure construction and cocatalyst methods [30,31,32,33,34]. 

## 2. Zinc Oxide Doped with Transition Metal Ions

In recent years, scholars have mainly studied the doping of aluminum, nickel, iron, manganese, copper and other substances [35,36] on zinc oxide. 

### 2.1. Doped with Divalent Metal Ions

**Figure 2:**
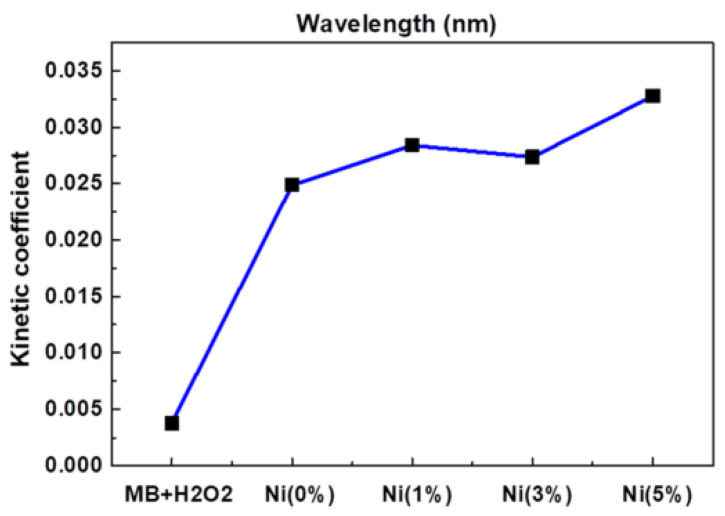
Kinetic constants of reaction between MB and ZnO doped with different Ni concentrations.

Jannat Hammouche et al. [37] prepared nickel-doped zinc oxide by the sol-gel method, which controlled the content of nickel at 0~5%, then annealed it and coated the prepared nickel-zinc oxide spin onto the silicon nanowires to obtain zinc oxide-nickel/silicon nanowires. The effects of nickel doping on the structure, morphology and photocatalytic properties of ZnO-Ni/Si nanowires were studied. Photocatalytic studies showed that nickel doping slightly promoted the photocatalytic degradation of methylene blue, and the effect was the best when the amount of nickel doping was 5%, as Figure 2 shows.

Lanqin Tang et al. [38] prepared zinc oxide photocatalysts with different co-doping degrees by the one-step dissolution method. It was found that the morphology of co-doped zinc oxide depended on the reaction temperature and the content of cobalt in solution. Additionally, it was found that the photocatalytic activity of cobalt-doped zinc oxide decreased, which might be due to the fact that the radius of cobalt and zinc ions are close to each other, thus forming a deep energy band gap level and reducing the photocatalytic activity. This indicates that doped transition metal ions are a double-edged sword, and further work can focus on the effects of continuous doping of different metal ions and their structures on the photocatalytic activity of zinc oxide [39], as Figure 2 shows.

### 2.2. Doped with Trivalent Metal Ions

**Figure 3:**
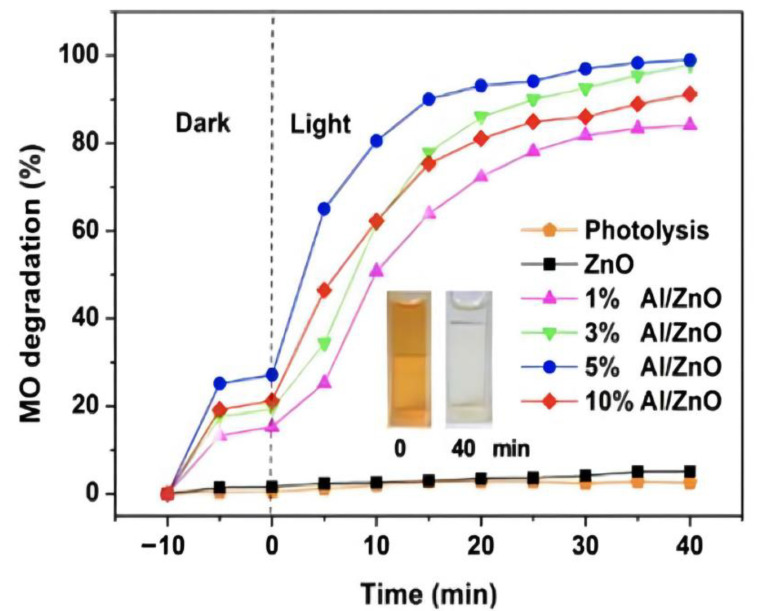
Photocatalytic efficiency of aluminum doping with different content (inset shows the color change in MO at time intervals 0 and 40 min).

Piangjai Peerakiatkhajohn et al. [40] used the sol-gel method to prepare low calcination temperature zinc oxide nanosheets and visible light-responsive Al-doped zinc oxide nanosheets. The results showed that calcination temperature and al doping had significant effects on the morphology, absorption properties and photocatalytic activity of zinc oxide nanoparticles. FE-SEM analysis showed that the spherical zinc oxide particles were transformed into nanorods and nanosheets at different calcination temperatures (200, 300 and 400 °C). At the same time, XRD analysis confirmed that the calcined zinc oxide nanosheets presented a hexagonal wurtzite structure, and with the increase of al doping amount, the absorption spectrum of al/zinc oxide nanosheets showed a red shift and the band gap became narrow, compared with the undoped zinc oxide nanosheets; it had excellent photocatalytic activity. The result showed that al doping can improve the photocatalytic activity of zinc oxide, as Figure 3 shows.

Sabrina Roguai et al. [41] successfully prepared fe-containing zinc oxide nanoparticles by the chemical coprecipitation method. The method was low-cost and easy to realize. The structure of the synthesized zinc oxide powders was characterized by XRD, the results showed that the synthesized zinc oxide powders are of hexagonal lead-zinc ore structure. The SEM images showed that the size of nanoparticles was not uniform and agglomerate, and the grain size of doped sampled decreased with the increase of doping concentration. Pure zinc oxide nanopowders exhibit higher photocatalytic activity for the degradation of methylene blue dyes than iron-doped zinc oxide, as measured by photochemical properties [42]. Whether it is divalent metal ions or trivalent metal ions, the main thing to pay attention to being the concentration. Too low doping concentration has a poor effect and cannot improve the photocatalytic efficiency of zinc oxide to the maximum extent. If the concentration is too high, the second phase will appear in XRD detection and the internal structure of zinc oxide will be destroyed. Therefore, the concentration of doped metal ions is the part that needs to be measured many times to get the best ratio concentration [43,44,45].

### 2.3. Co-Doping of Metal Ions

In addition to single-doped metal ions, co-doped that more than two kinds of metal ions has become a popular research direction to improve the photocatalytic performance of zinc oxide.

Fatemeh Dabir et al. [46] prepared pure zinc oxide films and zinc oxide films doped with aluminum and copper by the sol-gel method, and studied their electro-optical properties. The experimental results showed that the size of Al and Cu-doped zinc oxide nanosheets had increased, and formed the cross-linked nanostructured film. At the same time, it was found that the substitution and gap filling of Al and Cu dopants led to instability of the zinc oxide crystal structure and to additional point defects, which have a significant effect on the photocatalytic properties and electrical conductivity of zinc oxide.

**Figure 4:**
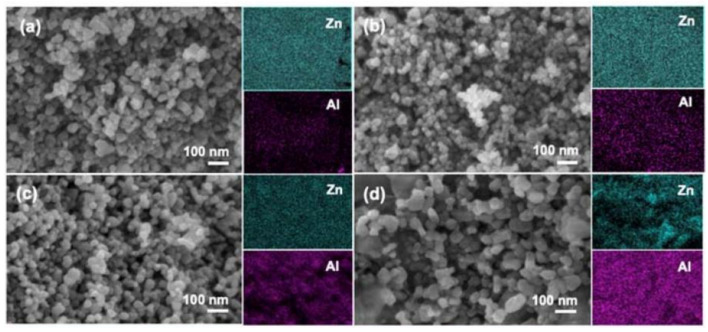
FE-SEM and elemental mapping images of: (**a**) 1% Al/ZnO; (**b**) 3% Al/ZnO; (**c**) 5% Al/ZnO; and (**d**) 10% Al/ZnO photocatalysts.

In addition, zinc oxide doped transition metal ions can also lead to the morphology of ZnO changed, such as doping aluminum can affect the internal stress of zinc oxide, cause the grain size of zinc oxide to be reduced and the specific surface area to be increased; it can also expose the more active site. As shown in Figure 4, abcd is, respectively, 1%, 3%, 5%, and 10% of Al doped ZnO in which it can be seen that the increase of Al content has a significant effect on the morphology of zinc oxide; nickel-doped zinc oxide particles were agglomerated into spheres, and copper and nickel-doped zinc oxide powders were reduced in size, agglomerated less, and dispersed better. These changes also affect the photocatalytic efficiency of zinc oxide. It is one of the effective ways to improve the photocatalytic efficiency of zinc oxide by doping metal ions.

## 3. Zinc Oxide Complex Precious Metal

### 3.1. Compounded with Precious Metal Silver

Silver is the most commonly used element in the precious metal compound zinc oxide, but it is also necessary to pay attention to the reduction of silver compound because of the decrease of the contact between silver and zinc oxide due to the accumulation of silver. The preparation of silver-loaded ZnO composite nanoparticles with nano-zinc oxide as precursor has been widely used in the field of photocatalysis. The addition of silver increases the size of the whole particle, and it can be judged by XRD that silver “Adheres” to the surface of nano-zinc oxide when the mass fraction of silver is small. After successfully loading silver, the absorption of uv-visible light of nano-zinc oxide is enhanced, which plays an important role in enhancing the photocatalytic efficiency. At present, as a mature technology, nano-zinc oxide supported silver has been widely used in photocatalysis and silver antibacterial and other fields, and achieved excellent results.

GEORGEKUTTY et al. [47] prepared Ag/zinc oxide powders by the anhydrous sol-gel method. The photocatalytic degradation experiments showed that the degradation rate of Rhodamine 6G was five times faster than that of pure zinc oxide under sunlight with the proper concentration of silver. It was also found that the photocatalytic activity decreased when the calcination temperature was higher than 400 °C, indicating that Ag/zinc oxide may have a more reasonable surface area at a lower temperature. However, the photocatalytic activity decreases with the increase of Ag content, which indicates that the Ag with high Ag content accumulates and forms photogenerated electron–hole pair complex center, thus decreasing the photocatalytic activity.

Liu et al. [48,49] prepared Ag/zinc oxide by hydrothermal and ultrasound-assisted methods, and used a polyol process to prepare silver nanowires and exhibited better photocatalytic activity than pure zinc oxide in photocatalytic experiments. The rate of methyl blue degradation increased by almost 25 times.

**Figure 5:**
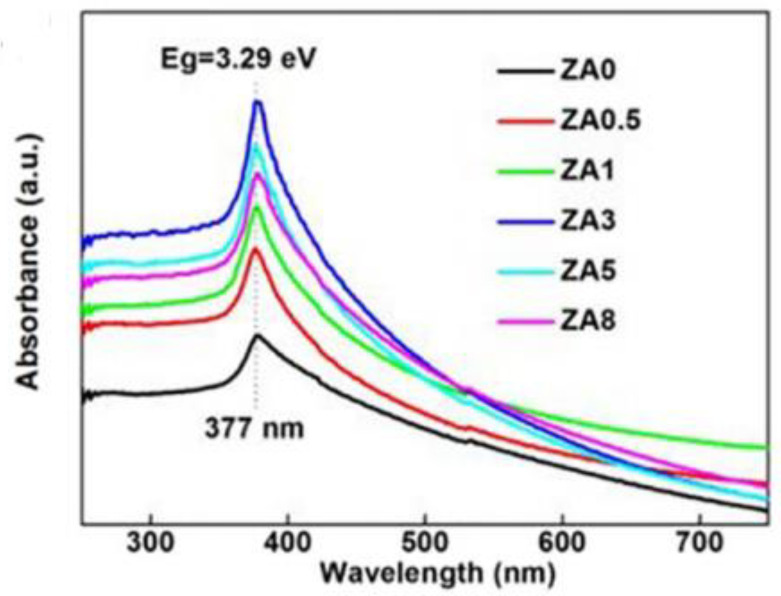
Absorption spectra of zinc oxide combined with different contents of silver.

Yutong Liu et al. [50] used a two-step polymer network gel method to synthesize silver-zinc oxide nanocatalysts, which were characterized to be uniformly distributed as compared with pure zinc oxide nanocatalysts; the specific surface area was large and the surface oxygen vacancy was abundant. When the silver content was 3%, the photocatalytic activity was the best, as shown in Figure 5, when the silver content was 3%, the absorption capacity of visible light was the strongest. It was speculated that the uniform dispersion of silver nanosheets and the increase of oxygen vacancies on the surface of zinc oxide nanoparticles are the main reasons for improving the photocatalytic efficiency. The strong interaction between silver and zinc oxide and surface oxygen vacancy enhanced the UV-VIS absorption, promoted the separation of photogenerated electrons and holes, and improved the photocatalytic performance.

### 3.2. Compound with Other Precious Metal

**Figure 6:**
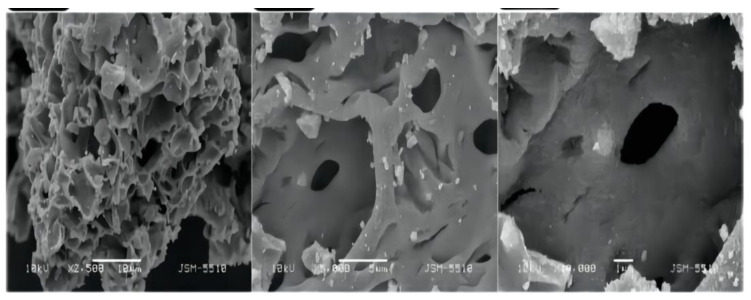
Morphology of zinc oxide afterAu composite.

Boyan Peychev et al. [51] used a novel starch-based solid solution method to prepare zinc oxide and gold-doped zinc oxide and synthesized nano-composite samples with different gold content. It was found that the nano-zinc oxide modified with gold nanocrystals had a great effect on the porosity of the nanocomposites, and the nanocomposites formed fewer micropores and mesoporous pores as shown in Figure 6, thus reducing the specific surface area, the photocatalytic activity decreases with the increase of gold content, and the photocatalytic activity was comparable to that of pure zinc oxide when the gold content is 0.05%.

## 4. Zinc Oxide Composite Semiconductor

### 4.1. Commonly Used Semiconductor Composite

**Figure 7:**
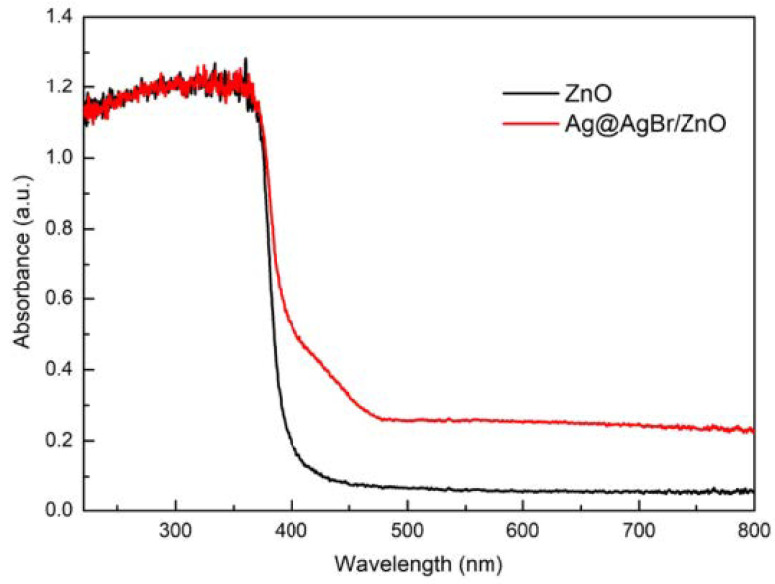
Comparison of UV-vis diffuse reflectance spectra before and after recombination.

Chong Tan et al. [52] synthesized Ag@AgBr/zinc oxide modified photocatalyst with a simple one-step hydrothermal method, and characterized the composite materials by various analytical methods, and analyzed the effect of Ag@AgBr/zinc oxide system on its photocatalytic performance. Results of UV-VIS diffuse reflectance spectrum clearly confirm that the red shift of absorption edge was transferred to visible spectral regionas showed in Figure 7. PL spectroscopic results showed that Ag@AgBr/zinc oxide had a lower electron–hole recombination ratio, which might be the reason why the photocatalytic activity of Ag@AgBr/zinc oxide composite was better than that of pure zinc oxide. The photocatalytic activity test results show that Ag@AgBr/zinc oxide modified catalyst had a significantly higher photocatalytic activity under visible light.

**Figure 8:**
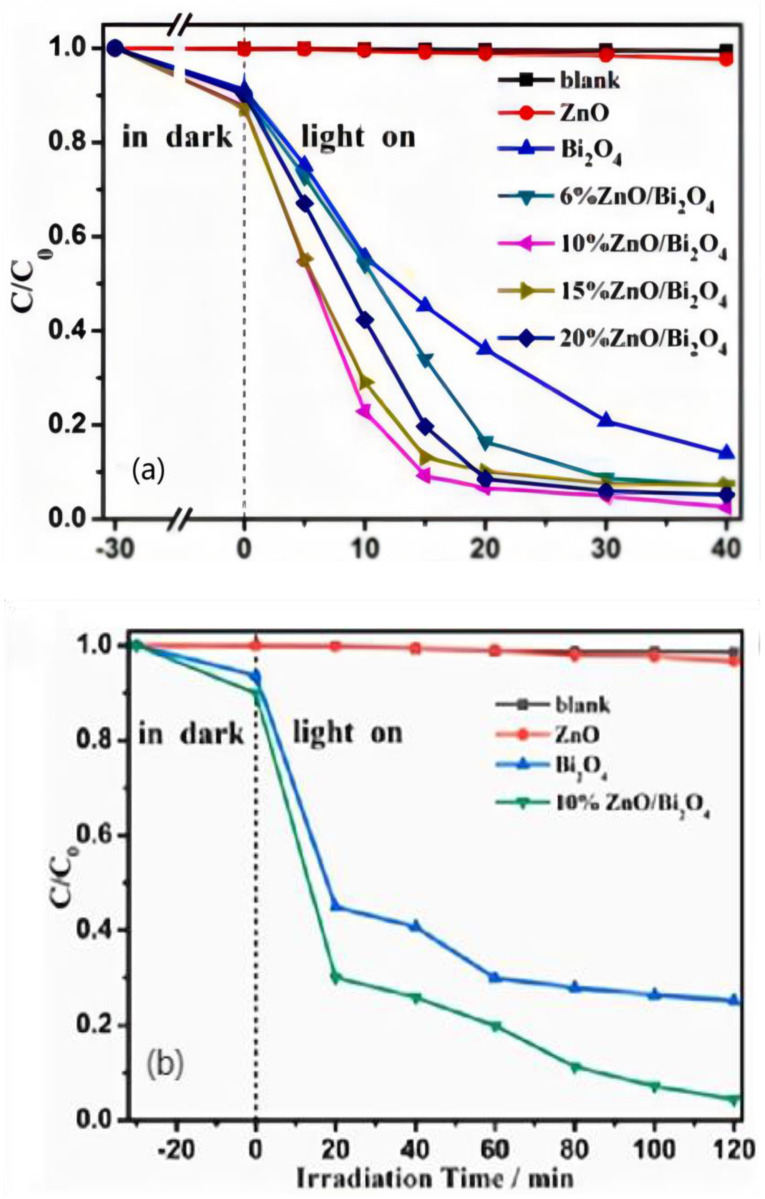
(**a**): Photocatalytic performances of the samples for degradation of CIP; (**b**): Photocatalytic degradation curves of MO solution over different photocatalysts under visible light irradiation.

Danping Wu et al. [53] successfully synthesized zinc oxide/Bi_2_O_4_ heterojunctions with different proportions by the one-step hydrothermal method. The results showed that 10% zinc oxide/Bi2O4 had the best photocatalytic degradation efficiency of methyl orange and ciprofloxacin, which was 97.5% and 95.6% as shown in Figure 8, respectively, 40.7 and 28.3 times of pure zinc oxide. Various characterization techniques and photoelectrochemical tests demonstrated that the enhanced photocatalytic activity was due to the formation of heterojunctions between zinc oxide and Bi_2_O_4_ with low charge transfer resistance and effective electron–hole pair separation.

Polat Gonullu Meryem et al. [54] prepared monolayer and bilayer zinc oxide/aluminium oxide thin films on glass substrates by ultrasonic spray pyrolysis. Through the degradation experiment of methylene blue solution, compared with pure zinc oxide film doped with aluminum ions, the photocatalytic ability of zinc oxide/alumina monolayer bilayer film was proved, and the photocatalytic activity could be further improved by designing the film.

In summary, the concentration, specific surface area and morphology of the composite semiconductors are important factors affecting the photocatalytic activity of zinc oxide composite semiconductors [55,56].

### 4.2. Graphene Composite

**Figure 9:**
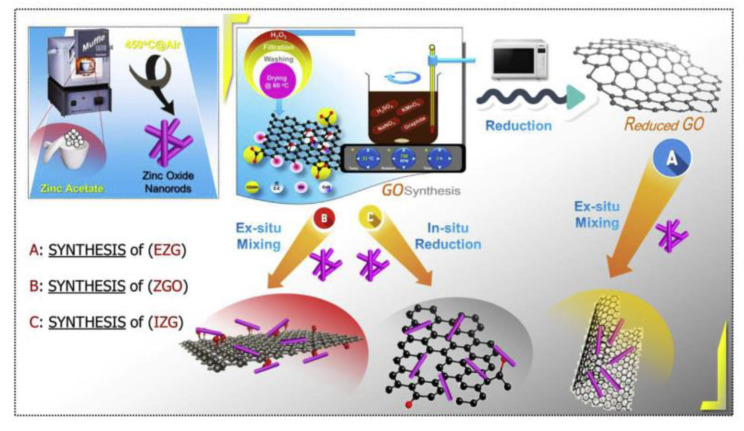
The synthesis of zno nanorods, go reduced go and their nanocomposites.

Since the discovery of graphene in 2004, the two-dimensional honeycomb-like lattice structure of graphene, which consists of a single layer of carbon atoms packed tightly together, had been characterized by its excellent electrical, thermal and mechanical properties, by the majority of scientific research workers of great concern [57,58,59,60]. Materials such as zinc oxide are considered to be an excellent material for pollutant degradation because of good carrier properties and high electron mobility. Because of its relatively large surface area as a catalyst, the graphene composite is able to absorb more pollutants, and its excellent electron mobility and carrier properties facilitate the transfer and separation of light-excited charges; graphene semiconductor composites are considered to have great potential to further improve the list of semiconductor materials of low visible light utilization and the high probability of excited electron–hole recombination; the synthesis of zno nanorods, go, reduced go, and their nanocomposites is shown in Figure 9. It is due to the introduction of graphene that the hole–electron recombination efficiency of the graphene/zinc oxide composite is reduced, while the active site is increased to increase the photocatalytic efficiency [61].

**Figure 10:**
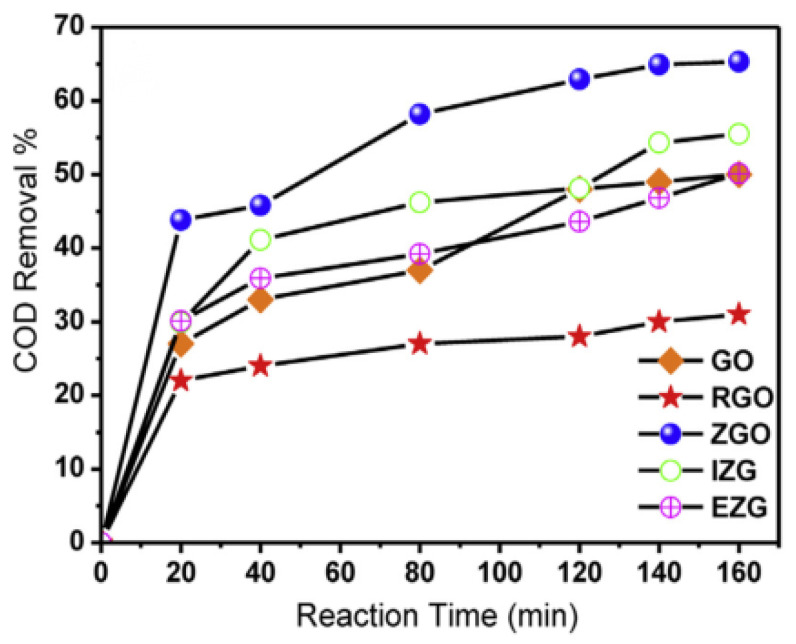
Demineralization of synthetic wastewater by different composite materials.

Ahmed F. Ghanem et al. [62] decorated the graphene surface with 5% zinc oxide nanorods and evaluated the demineralization of synthetic wastewater by chemical oxygen demand, the photocatalytic activity of the obtained nanocomposite was 30% and 35% higher than that of pure reduced and reduced graphene oxide as Figure 10 shown. This increase in efficiency can be attributed to the synergy between the planar structure of zinc oxide and graphene, resulting in the development of an unprecedented polycrystalline structure.

## 5. Changes in the Structure of Zinc Oxide

The morphology of zinc oxide will change with the doping of different metal elements or the composite of other semiconductors, and the change of morphology also has a certain impact on the photocatalytic performance of zinc oxide [63,64]. 

### 5.1. Doped with Transition Metal Ions

**Figure 11:**
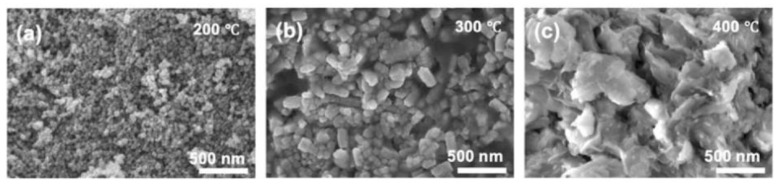
Particle size of zinc oxide at different calcination temperatures. (**a**) 200 °C, (**b**) 300 °C, (**c**) 400 °C.

The average particle size and its standard deviation and specific surface area of ZnfO NPs are shown in Figure 11. The results show that the particle size of ZnO NPs increased with increased calcination temperatures, the increase of particle size leads to the reduction of specific surface area, resulting in the reduction of light absorption capacity, and thus the reduction of photocatalytic efficiency [29].

### 5.2. Doped with Trivalent Metal Ions

**Figure 12:**
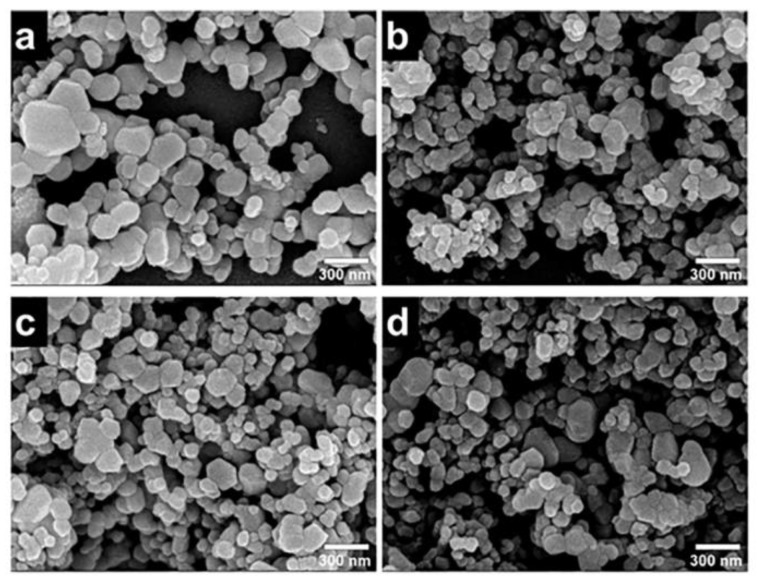
SEM images of Ag-ZnO photocatalysts: (**a**) [Ag^2+^]:[Zn2^+^] = 0, (**b**) [Ag^2+^]:[Zn2^+^] = 1, (**c**) [Ag^2+^]:[Zn2^+^] = 3, and (**d**) [Ag^2+^]:[Zn2_+_] = 5.

The SEM micrographs and particle size distributions of ZnO and Ag-ZnO nanoparticles are shown in Figure 12. As we can clearly see from Figure 12a–d that the average particle size of the as-prepared Ag-ZnO na-noparticles decreases in comparison with pure ZnO, when Ag ion sources was introduced during the second step of preparation process [36].

### 5.3. Composite Other Semiconductor

**Figure 13:**
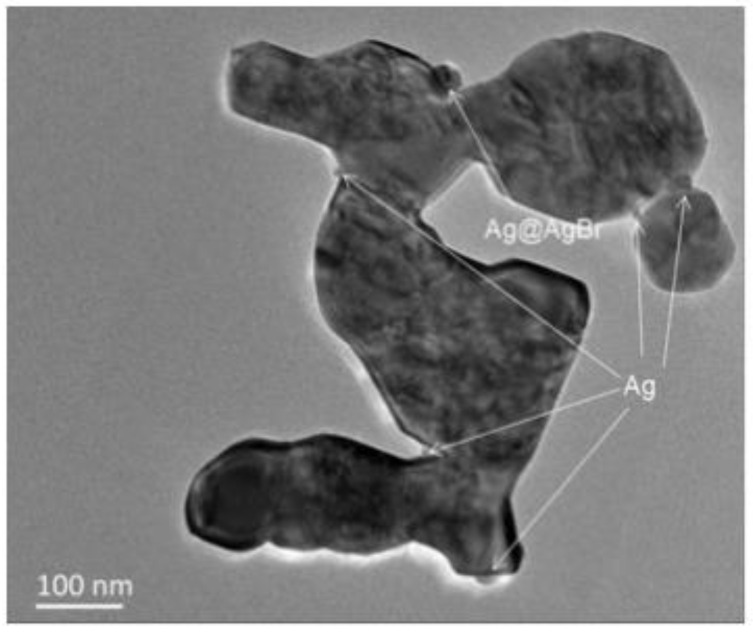
TEM image of Ag@AgBr/ZnO.

TEM images of Ag@AgBr/ZnO composites are presented in Figure 13. Figure 13a shows a partially porous sheet structure of an Ag@AgBr/ZnO sample, and small-sized spherical nanoparticles formed by Ag accumulation on the edge of the sheet can be observed. It is well known that smaller size Ag nanoparticles have a better effect on the enhancement of ZnO photocatalytic performance [41].

## 6. Conclusions and Outlook

### 6.1. Summary of Proposed Processes

The above three methods can improve the photocatalytic efficiency of zinc oxide to a certain extent, but the ways of realization are not completely the same. In addition, the commonness of the three methods lies in that when doping or recombining zinc oxide, the concentration should be carefully controlled. Different concentrations may cause damage to electronic holes or affect the morphology of zinc oxide, leading to a decline in the photocatalytic efficiency of zinc oxide.

1. Dope with transition metal ions

The doping of transition metal ions in zinc oxide is usually thought of as a metal site that can serve as a trap point for receiving photogenerated electrons or holes of a semiconductor and inhibit the recombination of carriers, thereby improving the photocatalytic activity of the semiconductor [65];

2. Complex precious metal

The composite of zinc oxide and precious metal can effectively separate electrons and holes, thus improving photocatalytic activity. Compared with other materials, the precious metals show higher structural controllability and chemical stability, therefore the noble metal compound zinc oxide is a reliable way to improve the photocatalytic efficiency of zinc oxide;

3. Composite other semiconductor

Combining zinc oxide with other semiconductors can not only increase the absorption of visible light region, but also reduce the recombination of carriers, so as to improve the photocatalytic activity. Due to the semiconductor between the conduction band of different energy levels, the photoexcited electrons can be rapidly separated from one semiconductor to the adjacent semiconductor, thus accelerating the separation of electron–hole pairs. 

### 6.2. Further Improvements on Zinc Oxide Structuring

Because these three methods improve the photocatalytic activity of zinc oxide through different methods, this paper proposes several ideas to further improve the photocatalytic efficiency of zinc oxide.

1. In the field of semiconductor composites, binary composites are widely used, while ternary composites are seldom studied. Recently, some researchers [66,67] have made ternary complexes of these ZnO binary complexes, these ternary complexes show higher stability, and the separation efficiency and separation time of electron–hole pairs are greatly increased, the photocatalytic activity is higher. This is due to the excitation of the narrow-band-gap semiconductors to the electron–hole pairs in the ternary complex under the irradiation of light, and because the conducting band energy of the narrow-band-gap semiconductors is lower than that of the other two semiconductors, therefore, the electrons are transferred from the narrow band-gap semiconductor to the wide band-gap semiconductor, and the valence band energy of the narrow band-gap semiconductor is greater than the valence band energy of the other two semiconductors, the holes in the valence band of narrow-band-gap semiconductors do not move or move to the valence band of wide-band-gap semiconductors, thus the effective separation of electron–hole pairs is realized. Therefore, the ternary complex is a feasible method to improve the photocatalytic efficiency of zinc oxide, and more attempts can be made in this area in the future;
2. On the basis of binary composite zinc oxide/semiconductor, doping of transition metal ions or modification of precious metals [68,69], that is, on the basis of heterojunction between zinc oxide and other materials, doping other transition metal ions or precious metals which can improve the photocatalytic efficiency of zinc oxide, the separation efficiency of photogenerated electron–hole pairs can be increased by increasing the specific surface area, so as to further improve the photocatalytic efficiency of zinc oxide.

The two methods mentioned above are the directions of developing new and better zinc oxide photocatalysts, which need to be tested and further discussed. In addition, there is still a better development space for the recovery of photocatalysts, such as the literature [70], which provides us with a new idea to separate the catalyst from the solution, in the follow-up experiments, the problems of catalyst recovery and catalyst stability can be solved on the basis of effectively improving the photocatalytic activity of zinc oxide to improve the possibility of industrial production.
